# Endoscopic duodenal perforation: surgical strategies in a regional centre

**DOI:** 10.1186/1749-7922-9-11

**Published:** 2014-01-24

**Authors:** Richard C Turner, Christina M Steffen, Peter Boyd

**Affiliations:** 1 Department of Surgery, Hobart Clinical School, University of Tasmania, Hobart, Australia; 2 Department of Surgery, Cairns Base Hospital, Cairns & Hinterland Health Service District, Cairns, Australia

**Keywords:** Duodenum, Perforation, Endoscopy, Surgery, Necrosis

## Abstract

**Background:**

Duodenal perforation is an uncommon complication of endoscopic retrograde cholangio-pancreatography (ERCP) and a rare complication of upper gastrointestinal endoscopy. Most are minor perforations that settle with conservative management. A few perforations however result in life-threatening retroperitoneal necrosis and require surgical intervention. There is a relative paucity of references specifically describing the surgical interventions required for this eventuality.

**Methods:**

Five cases of iatrogenic duodenal perforation were ascertained between 2002 and 2007 at Cairns Base Hospital. Clinical features were analyzed and compared, with reference to a review of ERCP at that institution for the years 2005/2006.

**Results:**

One patient recovered with conservative management. Of the other four, one died after initial laparotomy. The other three survived, undergoing multiple procedures and long inpatient stays.

**Conclusions:**

Iatrogenic duodenal perforation with retroperitoneal necrosis is an uncommon complication of endoscopy, but when it does occur it is potentially life-threatening. Early recognition may lead to a better outcome through earlier intervention, although a protracted course with multiple procedures should be anticipated. A number of surgical techniques may need to be employed according to the individual circumstances of the case.

## Background

Duodenal perforation is an uncommon complication of endoscopic retrograde cholangiopancreatography (ERCP) and a very rare complication of upper gastrointestinal endoscopy. Most series report a majority of non-life-threatening perforations which settle with conservative management [1,2]. There are few references specifically describing the surgical interventions required for the minority of iatrogenic duodenal perforations where surgery is indicated.

Five cases of iatrogenic duodenal perforation occurring between 2002 and 2007 at Cairns Base Hospital are presented for comparison, with reference to a review of ERCP at Cairns Base Hospital for the years 2005/2006. Further, a focused review of the literature was undertaken to inform discussion of the surgical management of such cases.

## Methods

Cairns Base Hospital is a secondary referral hospital in Far North Queensland, Australia. It serves a catchment population of approximately 250 000, 15% of which identify as Indigenous Australian. Hospital surgical audit and endoscopy records for the period 2002–2008 were searched for cases of duodenal perforation following endoscopy or ERCP. Age, sex, indication for endoscopy/ERCP, timing or delay to diagnosis and definitive management, type of perforation, surgical management, complications, length of stay, and late morbidity were recorded for each case.

An audit of ERCP at Cairns Base Hospital for the two year period 2005/2006 was utilized to determine incidence of complications of ERCP and is presented in Tables 1 and 2.

**Table 1:** Complications of ERCP procedures for 2005–6 at Cairns Base Hospital (N = 211)

**Complication**	**N (%)**
Pancreatitis	9 (4.3%)
Cholangitis	7 (3.3%)
Bleeding	4 (1.9%)
Perforation	2 (0.95%)
Death	3 (1.4%)
Other: Stroke	1 (0.5%)
**Total (with complications)**	22 (12.3%)

Adapted from Cotton et al. 1991 [3].

**Table 2:** Indications for ERCP 2005–06, Cairns Base Hospital (N = 202)

**Indication**	**N (%)**
CBD stone (s)	115 (57%)
Cholangitis	6 (3%)
Malignant jaundice	29 (14%)
Stent change or unblocking	33 (16%)
Abdominal pain, abnormal LFTs, dilated duct	5 (2.5%)
Chronic pancreatitis	10 (10%)
Abnormal CT	1 (0.5%)
Bile leak	3 (1.5%)

For the focused literature review, a PubMed search was undertaken using the terms “duodenal perforation”, “endoscopic” and “retroperitoneal necrosis”. Case-based articles cited by reviews were secondarily sourced. Articles with English language abstracts were considered, and excluded if endoscopy was not the cause of the perforation (rather a treatment) or if specific operative details were not reported. Similarly, only cases that underwent some form of surgical management were included.

Approval to access and analyze de-identified patient records for this study was given by the Human Research Ethics Committee of the Cairns and Hinterland Health Service District.

## Results

**Figure 1:**
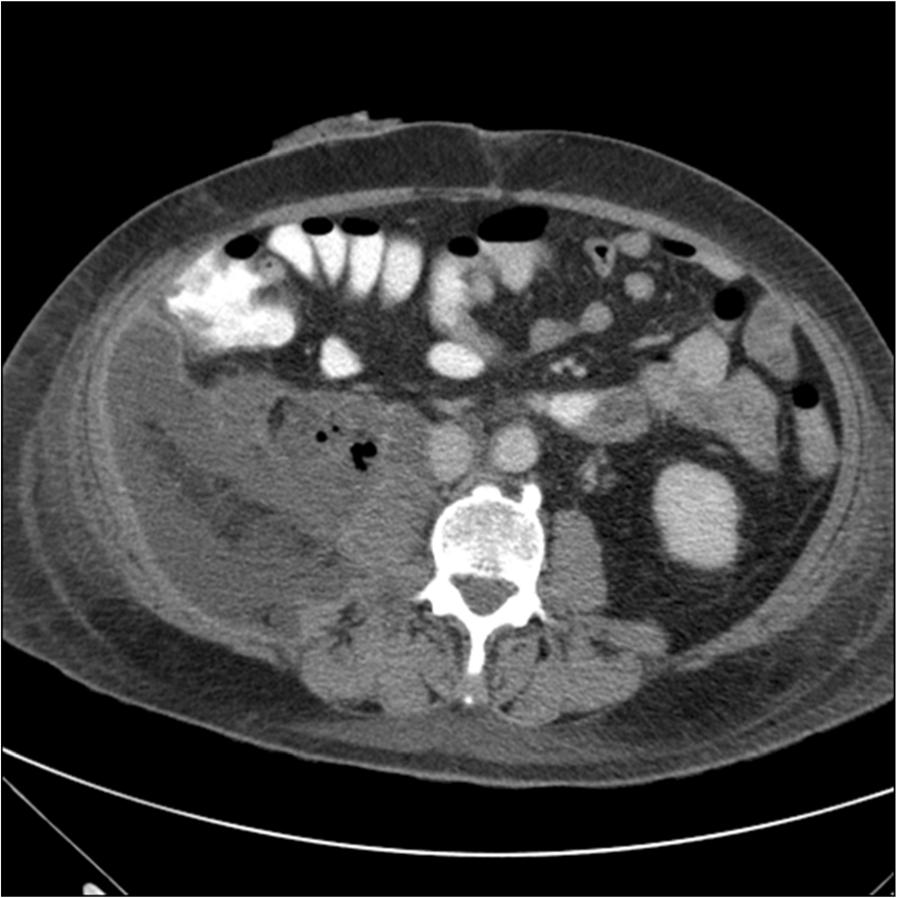
CT image showing extensive retroperitoneal necrosis prior to surgical intervention (Case 2).

**Figure 2:**
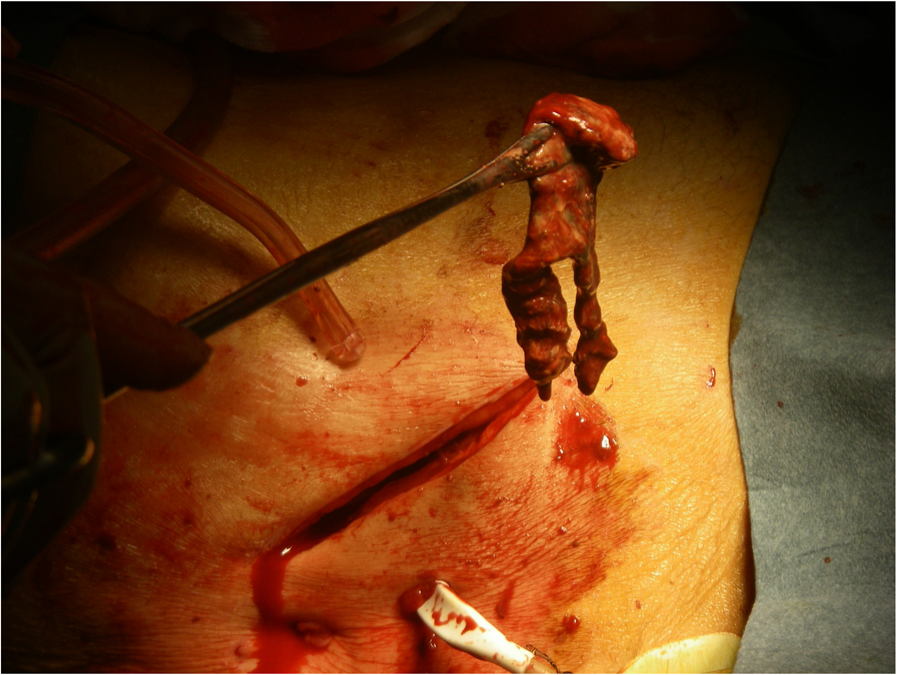
Necrotic retroperitoneal tissue debrided via right flank incision (Case 1).

Five patients sustaining iatrogenic duodenal perforation were identified. The clinical data pertaining to these are presented in Table 3. All four of the ERCP cases had an associated pre-cut sphincterotomy. No significant bleeding was noted, and no additional procedures such as lithotripsy or stenting were performed. In two cases, there was no specific evidence of choledocholithiasis, with the ERCP being intended solely for diagnostic purposes. Figure 1 shows a representative CT image from Case 2 prior to surgical intervention. Figure 2 illustrates the necrotic retroperitoneal material debrided via a right flank incision in Case 1.

**Table 3:** Characteristics of endoscopically induced duodenal injuries, Cairns Base Hospital, 2002–2008

**Case (year)**	**1 (2002)**	**2 (2004)**	**3 (2005)**	**4 (2006)**	**5 (2007)**
**Age/Sex**	**51 male**	**69 male**	**42 female**	**61 female**	**72 male**
Indication for ERCP/endoscopy	Post-cholecystectomy pain	Choledocholithiasis	Post- cholecystectomy pancreatitis	Choledocholithiasis	Post-cholecystectomy pain
Post-procedure symptoms, signs	Severe abdominal pain, tachycardia	Severe abdominal pain	Mild abdominal pain	Abdominal pain	Abdominal pain
Type of perforation	Not identified	Not identified (Duodenal diverticulum)	Type 2 (see Results)	Not identified	Type 1 (see Results) (Duodenal diverticulum)
Delay to Diagnosis/Intervention	48 hours then 5 weeks	5 days	Immediate diagnosis	Immediate diagnosis, surgery within 24 hours	Immediate diagnosis, surgery at 6 hours
Indications for surgery	a) Duodenal perforation	a) Duodenal perforation	Nil	a) Duodenal perforation	a) Large defect duodenum,
a) at diagnosis	b) Infected retroperitoneal necrosis/collections	b) Extensive retroperitoneal necrosis/collections Persistent duodenal leak			b) Extensive retroperitoneal necrosis/collections
b) subsequent	Duodenal stenosis, Necrosis of posterior caecal wall			b) Extensive retroperitoneal necrosis	a) Laparotomy, repair duodenum
Management	a) Laparotomy	a) Laparotomy	Conservative	a) Laparotomy, retroperitoneal washout, pyloric, exclusion, gastrojejunostomy, jejunal feeding tube	b) Open drainage/evacuation right retroperitoneal space x 2
a) on diagnosis	b) Attempted percutaneous drainage	b) 7 x debridement of necrosis	(no surgery)		Drainage right scrotum
b) subsequent	2 x Open drainage procedure right retroperitoneal space	Open drainage right inguinoscrotal tract			
	Right hemicolectomy, end ileostomy and mucous fistula	Pyloric exclusion, gastrojejunostomy			
Complications of treatment	Deep vein thrombosis	Gastroparesis, UTI, CVL infection, wound infection, left brachial plexopathy	Nil	Necrotising fasciitis right thigh/abdomen	Right inguinal haematoma
					Incisional hernia
					Seroma
Length of stay (days)	99	132	4	6	63
Case fatality	No	No	No	Yes	No
Residual disability	Residual presacral collection and sinus to right iliac fossa	Retained CBD stones removed 2007	Nil	Died	Nil

In cases 1, 2 and 4, the actual duodenal perforation could not be identified at operation. This may have been due to a smaller size of the perforation and/or delay to surgery resulting in difficulty identifying the perforation. Ongoing leakage in Case 2 necessitated subsequent pyloric exclusion and gastrojejunostomy. Case 5, where endoscopy alone was performed, is likely to have perforated through a duodenal diverticulum, which is a known risk factor for perforation both in endoscopy and ERCP [4-6]. This large perforation was obvious at the time and early operation enabled definitive repair. As integrity of the repair was demonstrated radiologically, the subsequent delayed extensive retroperitoneal necrosis presumably arose from the leakage that occurred in the few hours between injury and laparotomy for repair.

Timing of intervention was assisted by serial computerized tomography examination. In the four cases treated surgically, definitive intervention consisted of open surgical drainage with or without subsequent CT-guided percutaneous drainage of amenable collections. While open surgical drainage was immediately effective in all cases, percutaneous drainage as an initial intervention was not effective in Case 1, attributable to the large volumes of semi-solid necrotic material in the retroperitoneum of this patient. This is consistent with experience in pancreatic necrosectomy [7,8]. In contrast, percutaneous drainage was an effective modality for the smaller, less accessible but more fluid presacral collection in Case 5.

Retroperitoneal necrosis was progressive and in most cases multiple operations were required due to ongoing symptoms. An oblique right flank to right iliac fossa incision was performed in Cases 1 and 5 giving good access to the upper and lower right retroperitoneal space and to the presacral space. A feature of the three cases in males was involvement of the right inguinoscrotal tract, with Cases 2 and 5 requiring separate drainage of symptomatic inguinoscrotal collections. None had pre-existing hernias.

One patient (Case 4) died indirectly as a result of the perforation, from sepsis associated with vascular access. This patient had significant co-morbidities, being steroid-dependent for pulmonary interstitial fibrosis and rheumatoid arthritis. Of the four survivors, one recovered quickly with conservative management alone, but the other three endured long hospital stays, underwent multiple surgical and other procedures, and developed short-term and long-term complications as a result of the original perforation and its treatment.

## Discussion

All cases in this series were managed by General Surgeons at a regional hospital, serving a population of 250 000 and geographically remote from larger facilities. The endoscopic procedures were performed by a Gastroenterologist and a General Surgeon, both of whom were formally trained and accredited in these skills. As upper endoscopy and now ERCP are readily available in larger regional centres, an awareness of this serious but fortunately rare complication and its clinical course is useful for General Surgeons faced with its management. Certainly Case 5, undertaken with the benefit of specific experience gained in the management of Case 1, does seem to have had a better quality outcome, with shorter length of stay, fewer procedures, and fewer complications.

While duodenal perforation at endoscopy alone is extremely rare, the rate during ERCP is significantly higher, estimated to be between 0.4 and 1% [9]. The rate of 0.95% in the audited series from Cairns Base Hospital is within these limits (Table 1). The indications for ERCP at our institution are shown in Table 2. It should be noted that two patients in the series had the uncommon indication of post-cholecystectomy pain. During the time period of this series, no other imaging modalities for the common bile duct were readily available. Despite the excellent standards set for training and quality assurance, ERCP, particularly when associated with sphincterotomy, still incurs a definite risk of complication, and its indications should be primarily interventional [10]. The emerging availability in regional centres of less invasive diagnostic modalities such as MRCP and endoscopic ultrasound (EUS) should reduce exposure to the risk of duodenal perforation in this group, [11,12] as has indeed been the case at our institution since 2007. Where these are not available, consideration should be given to transferring patients to centres where they are, particularly when there is no therapeutic intent at the outset.

Four types of duodenal perforation have been described – Type 1: lateral duodenal wall, Type 2: peri-Vaterian duodenum, Type 3: bile duct, and Type 4: tiny retroperitoneal perforations caused by the use of compressed air during endoscopy. Most perforations are Type 2, due to concomitant endoscopic sphincterotomy, and may be suitable for a trial of conservative management [13-15]. In our series, Case 3 was documented as a Type 2 perforation. Case 5 was documented as a Type 1 perforation, and Cases 1, 2, 4 were most likely this, based on the ensuing clinical course. Type 1 perforations have the most serious consequences and typically require complex and invasive treatment. They are mostly caused by the endoscope itself and may result in considerable intra- or extraperitoneal spillage of duodenal fluid (a mixture of gastric juice, bile and pancreatic juice), the latter causing rapid, extensive, and ongoing necrosis of the right retroperitoneum. The patient becomes intensely catabolic with fevers, raised inflammatory markers, leucocytosis, and nutritional depletion. Without surgical intervention death is likely from a combination of massive auto-digestion, nutritional depletion and sepsis. Delay in diagnosis increases the likelihood of a fatal outcome [16,17].

Various management algorithms for duodenal injuries have been proposed, largely focusing on early diagnosis and the decision for surgical management [18-21]. Indications for surgery have been well described. If a Type 1 injury is noted at endoscopy or on subsequent imaging (eg. extravasation of contrast), immediate operative intervention is generally mandated. Failure of conservative management due to signs of progressive systemic inflammatory response syndrome (SIRS) is a relative indication for operation. Guidelines for specific operative strategies in the face of ERCP-related duodenal injury and retroperitoneal necrosis have been proposed, but are often based on evidence derived from individual case reports or case series, or from experience in the trauma setting [22,23]. Due to its uncommon nature, prospective comparative studies to determine the optimal procedure for endoscopically induced duodenal perforation have yet to be published [24].

Published case series and reports regarding possible surgical management options for endoscopically induced Type 1 and 2 duodenal injuries are summarized in Table 4[13,18,19,21,25-34]. In general, operative procedures are tailored to conditions encountered at the time of laparotomy, as well as to any underlying pathology that preceded or was the indication for the endoscopic procedure. Primary repair of a breach in the duodenal wall may be possible where the injury is diagnosed early and there is limited contamination of surrounding tissues. Kocherization is usually needed to facilitate this, along with debridement of any devitalized tissue. Additional operative variations worthy of consideration include repair in one or two layers, transverse or longitudinal closure, and augmentation with a jejunal serosal [35] or omental patch. For patients deemed to be at high risk for leak or fistula formation, a number of additional protective measures have been proposed [24,36]. Tube decompression involves placement of a trans-mural trans-parietal duodenostomy or jejunostomy tube [37]. There are concerns that this engenders additional trauma to the gastrointestinal tract and may not provide adequate decompression. Duodenal diverticulation is a complex procedure that involves duodenal repair, distal Billroth II gastrectomy, placement of a decompressive duodenostomy tube, and peri-duodenal drainage [38]. This is obviously time-consuming and is often inappropriate for haemodynamically unstable patients. A less onerous procedure is pyloric exclusion, which entails primary duodenal repair, pyloric suture or stapling via greater curvature gastrotomy, and gastrojejunostomy using the gastrotomy incision [39]. In certain circumstances, it may be suitable to perform a duodenojejunostomy, preferably with Roux-en-Y reconstruction [40]. Such a maneuver would obviously be predicated on a stable patient and a duodenum wall that is amenable to sutures. It is clear that the General Surgeon must have a variety of techniques in his/her repertoire in order to adapt to the situation at hand.

**Table 4:** Reports in the literature of Type 1 and 2 duodenal injuries caused by endoscopic procedures

**Case/series**	**N =**	**Range of management strategies for:**			**Average days in hospital**	**Case fatality (%)**
		**Duodenal injury**	**Retroperitoneal necrosis**	**Underlying pathology**		
Stapfer et al. 2000 [13]	8	Pyloric exclusion and gastro-jejunostomy	Drain placement	Cholecystectomy	62.9	2 (25%)
		Tube duodenostomy		CBD exploration		
		Duodeno-antrectomy		Hepatico-jejunostomy		
Preetha et al. 2003 [25]	13	Primary repair	Not described	Cholecystectomy	23.8	3 (23.1%)
		Pyloric exclusion and gastro-jejunostomy		CBD exploration		
		T-tube		Hepatico-jejunostomy		
		Bowel decompression				
Kalyani et al. 2005 [26]	1	Jejunal serosal patch	Not required	Nil required	>15	0 (0%)
Melita et al. 2005 [27]	1	Nil required	CT-guided abscess drainage	Nil required	Not specified	0 (0%)
Wu et al. 2006 [18]	10	Primary repair	Drain placement	Cholecystectomy	31.4	4 (40%)
		Omental patch	Open abscess drainage	CBD exploration		
		Duodenostomy	Percutaneous abscess drainage	Cholecysto-jejunostomy		
Fatima et al. 2007 [28]	22	Primary repair	Drain placement	Choledocho-jejunostomy	16	3 (13.6%)
		Omental patch				
Knudson et al. 2008 [29]	12	Primary repair	Drain placement	Hepatico-jejunostomy	4.5	0 (0%)
		T-tube	Open abscess drainage			
		Omental patch				
		Duodenostomy tube				
		Gastrostomy				
		Jejunostomy tube				
		Pyloric exclusion				
Mao et al. 2008 [30]	3	Nil required	Drain placement	Cholecystectomy	50	0 (0%)
				CBD exploration		
				T-tube		
Angiò et al. 2009 [31]	1	Kocherization and primary repair	Not described	CBD exploration	23	0 (0%)
Avgerinos et al. 2009 [19]	15	Primary repair	Not described	Choledocho-duodenostomy	42	3 (20%)
		Omental patch				
		Pyloric exclusion				
		Gastro-enterostomy				
Morgan et al. 2009 [32]	10	Primary repair gastrojejunostomy	Drain placement		Not available	1 (10%)
Dubecz et al. 2012 [33]	4	Primary repair	Not described	Hepatico-jejunostomy	23	0 (0%)
		T-tube				
Ercan et al. 2012 [21]	13	Primary repair	Percutaneous abscess drainage	Cholecystectomy	10.2	6 (46.2%)
		Pyloric exclusion	Open abscess drainage	CBD exploration		
		Gastro-enterostomy		T-tube		
Caliskan et al. 2013 [34]	9	Primary repair	Not described	CBD exploration	22.6	4 (44.4%)
		Duodenostomy		T-tube		
		Pyloric exclusion, gastro-jejunostomy		Pancreatico-duodenectomy		

The other important issue to contend with in duodenal injuries is the management of retroperitoneal necrosis or sepsis. In most cases where laparotomy is performed, some degree of debridement and placement of drains is undertaken. This may be all that can be done if primary duodenal repair is not feasible, or the perforation cannot be localized amid the devitalized tissue. As illustrated by our own case series, repeated drainage procedures are often necessary if signs of recurrent sepsis develop. As has been noted by other authors, [41] males are also at risk of developing sepsis of the inguinoscrotal tract. Percutaneous drainage of any recurrent collections may be attempted using radiological guidance, unless the semi-solid nature of the debris necessitates an open approach. The technique of video-assisted retroperitoneal debridement, [42] as validated for infected necrotizing pancreatitis, may be of use, but there have been no reports of its application in this context.

## Conclusion

Retroperitoneal necrosis due to duodenal perforation is a rare but serious complication of ERCP. Early recognition based on risk factors and clinical suspicion may lead to a better outcome, although a protracted course with multiple and various types of procedures should be anticipated. Urgent interventions typically involve debridement and drainage, duodenal repair where feasible, and if indicated, duodenal diversion or other protective procedures. Familiarity with a number of possible surgical strategies is desirable due to the need to adapt to individual circumstances. Surgical management plans should also take into account any underlying pathology that was the initial indication for the endoscopic procedure, although definitive procedures may not be feasible at first operation. The use of ERCP for purely diagnostic purposes should only be considered where less invasive imaging modalities are not possible.

## Abbreviations

CBD: Common bile duct; CVL: Central venous line; CT: Computerized tomography; ERCP: Endoscopic retrograde cholangiopancreatography; EUS: Endoscopic ultrasound; LFTs: Liver function tests; MRCP: Magnetic resonance cholangiopancreatography; SIRS: Systemic inflammatory response syndrome; UTI: Urinary tract infection.

## Competing interests

The authors declare that they have no competing interests.

## Authors’ contributions

RT contributed clinical cases to the series, co-wrote the manuscript and attended to reviewer comments. CS contributed clinical cases to the series and co-wrote the manuscript. PB provided the summary data of institutional endoscopy outcomes and edited the first draft of the manuscript. All authors read and approved the final manuscript.

## References

[B1] Enns R Eloubeidi MA Mergener K Jowell PS Branch MS Pappas TM Baillie J ERCP-related perforations: risk factors and management Endoscopy 2002 34 4 293 298 11932784 10.1055/s-2002-23650

[B2] Kayhan B Akdoğan M Sahin B ERCP subsequent to retroperitoneal perforation caused by endoscopic sphincterotomy Gastrointest Endosc 2004 60 5 833 835 15557971 10.1016/s0016-5107(04)02171-6

[B3] Cotton PBLG Vennes J Geenen JE Russell RC Meyers WC Liguory C Nickl N Endoscopic sphincterotomy complications and their management: an attempt at consensus Gastrointest Endosc 1991 37 3 383 393 2070995 10.1016/s0016-5107(91)70740-2

[B4] Christensen M Matzen P Schulze S Rosenberg J Complications of ERCP: a prospective study Gastrointest Endosc 2004 60 5 721 731 15557948 10.1016/s0016-5107(04)02169-8

[B5] Miller RE Bossart PW Tiszenkel HI Surgical management of complications of upper gastrointestinal endoscopy and esophageal dilation including laser therapy Am Surg 1987 53 11 667 671 3500661

[B6] Ames JT Federle MP Pealer KM Perforated duodenal diverticulum: clinical and imaging findings in eight patients Abdom Imaging 2009 34 2 135 139 18253777 10.1007/s00261-008-9374-x

[B7] Slavin JGP Sutton R Hartley M Rowlands P Garvey C Hughes M Neoptolemos J Management of necrotizing pancreatitis World J Gastroenterol 2001 7 4 476 481 11819813 10.3748/wjg.v7.i4.476 PMC4688657

[B8] Freeny PC Hauptmann E Althaus SJ Traverso LW Sinanan M Percutaneous CT-guided catheter drainage of infected acute necrotizing pancreatitis: techniques and results Am J Roentgenol 1998 170 4 969 975 9530046 10.2214/ajr.170.4.9530046

[B9] Habr-Gama A Waye JD Complications and hazards of gastrointestinal endoscopy World J Surg 1989 13 2 193 201 2658366 10.1007/BF01658399

[B10] Cotton PB Is your sphincterotomy really safe–and necessary? Gastrointest Endosc 1996 44 6 752 755 8979074 10.1016/s0016-5107(96)70068-8

[B11] Vandervoort J Soetikno RM Tham TC Wong RC Ferrari APJ Montes H Roston AD Slivka A Lichtenstein DR Ruymann FW Risk factors for complications after performance of ERCP Gastrointest Endosc 2002 56 5 652 656 12397271 10.1067/mge.2002.129086

[B12] Halme L Doepel M von Numers H Edgren J Ahonen J Complications of diagnostic and therapeutic ERCP Ann Chir Gynaecol 1999 88 2 127 131 10392249

[B13] Stapfer M Selby RR Stain SC Katkhouda N Parekh D Jabbour N Garry D Management of duodenal perforation after endoscopic retrograde cholangiopancreatography and sphincterotomy Ann Surg 2000 232 2 191 198 10903596 10.1097/00000658-200008000-00007 PMC1421129

[B14] Suissa A Yassin K Lavy A Lachter J Chermech I Karban A Tamir A Eliakim R Outcome and early complications of ERCP: a prospective single center study Hepatogastroenterology 2005 52 62 352 355 15816433

[B15] Williams EJ Taylor S Fairclough P Hamlyn A Logan RF Martin D Riley SA Veitch P Wilkinson ML Williamson PR Risk factors for complication following ERCP; results of a large-scale, prospective multicenter study Endoscopy 2007 39 9 793 801 17703388 10.1055/s-2007-966723

[B16] Bharathi R Rao P Ghosh K Iatrogenic duodenal perforations caused by endoscopic biliary stenting and stent migration: an update Endoscopy 2006 38 12 1271 1274 17163332 10.1055/s-2006-944960

[B17] Doerr RJ Kulaylat MN Booth FV Corasanti J Barotrauma complicating duodenal perforation during ERCP Surg Endosc 1996 10 3 349 351 8779077 10.1007/BF00187390

[B18] Wu HM Dixon E May GR Sutherland FR Management of perforation after endoscopic retrograde cholangiopancreatography (ERCP): a population-based review HPB (Oxford) 2006 8 5 393 399 18333093 10.1080/13651820600700617 PMC2020744

[B19] Avgerinos DV Llaguna OH Lo AY Voli J Leitman IM Management of endoscopic retrograde cholangiopancreatography: related duodenal perforations Surg Endosc 2009 23 4 833 838 18830749 10.1007/s00464-008-0157-9

[B20] Machado NO Management of duodenal perforation post-endoscopic retrograde cholangiopancreatography. When and whom to operate and what factors determine the outcome? A review article JOP 2012 13 1 18 25 22233942

[B21] Ercan M Bostanci EB Dalgic T Karaman K Ozogul YB Ozer I Ulas M Parlak E Akoglu M Surgical outcome of patients with perforation after endoscopic retrograde cholangiopancreatography J Laparoendosc Adv Surg Tech A 2012 22 4 371 377 22288879 10.1089/lap.2011.0392

[B22] Carrillo EH Richardson JD Miller FB Evolution in the management of duodenal injuries J Trauma Inj Infect Crit Care 1996 40 6 1037 1046 10.1097/00005373-199606000-00035 8656463

[B23] Degiannis E Boffard K Duodenal injuries Br J Surg 2000 87 11 1473 1479 11091233 10.1046/j.1365-2168.2000.01594.x

[B24] Lai CH Lau WY Management of endoscopic retrograde cholangiopancreatography-related perforation Surgeon 2008 6 1 45 48 18318088 10.1016/s1479-666x(08)80094-7

[B25] Preetha M Chung YF Chan WH Ong HS Chow PK Wong WK Ooi LL Soo KC Surgical management of endoscopic retrograde cholangiopancreatography-related perforations ANZ J Surg 2003 73 12 1011 1014 14632894 10.1046/j.1445-2197.2003.t01-15-.x

[B26] Kalyani A Teoh CM Sukumar N Jeiunal patch repair of a duodenal perforation Med J Malaysia 2005 60 2 237 238 16114169

[B27] Melita G Currò G Iapichino G Princiotta S Cucinotta E Duodenal perforation secondary to biliary stent dislocation: a case report and review of the literature Chir Ital 2005 57 3 385 388 16231831

[B28] Fatima J Baron TH Topazian MD Houghton SG Iqbal CW Ott BJ Farley DR Farnell MB Sarr MG Pancreaticobiliary and duodenal perforations after periampullary endoscopic procedures: diagnosis and management Arch Surg 2007 142 5 448 454 17515486 10.1001/archsurg.142.5.448

[B29] Knudson K Raeburn CD McIntyre RCJ Shah RJ Chen YK Brown WR Stiegmann G Management of duodenal and pancreaticobiliary perforations associated with periampullary endoscopic procedures Am J Surg 2008 196 6 975 982 19095118 10.1016/j.amjsurg.2008.07.045

[B30] Mao Z Zhu Q Wu W Wang M Li J Lu A Sun Y Zheng M Duodenal perforations after endoscopic retrograde cholangiopancreatography: experience and management J Laparoendosc Adv Surg Tech A 2008 18 5 691 695 18803511 10.1089/lap.2008.0020

[B31] Angiò LG Sfuncia G Viggiani P Faro G Bonsignore A Licursi M Soliera M Galati M Putortì A Management of perforations as adverse events of ERCP plus ES. Personal experience G Chir 2009 30 11–12 520 530 20109385

[B32] Morgan KA Fontenot BB Ruddy JM Mickey S Adams DB Endoscopic retrograde cholangiopancreatography gut perforations: when to wait! When to operate! Am Surg 2009 75 6 477 483 19545095

[B33] Dubecz A Ottmann J Schweigert M Stadlhuber RJ Feith M Wiessner V Muschweck H Stein HJ Management of ERCP-related small bowel perforations: the pivotal role of physical investigation Can J Surg 2012 55 2 99 104 22564521 10.1503/cjs.027110 PMC3310764

[B34] Caliskan K Parlakgumus A Ezer A Colakoglu T Törer N Yildirim S Surgical management of endoscopic retrograde cholangiopancreatography related injuries Hepatogastroenterology 2013 60 121 76 78 23841162

[B35] McInnes WD Aust JB Cruz AB Root HD Traumatic injuries of the duodenum: a comparison of primary closure and the jejunal patch J Trauma 1975 15 847 853 1177330

[B36] Jansen M Du Toit DF Warren BL Duodenal injuries: surgical management adapted to circumstances Injury 2002 33 7 611 615 12208065 10.1016/s0020-1383(02)00108-0

[B37] Stone HH Fabian TC Management of duodenal wounds J Trauma 1979 19 334 339 448769 10.1097/00005373-197905000-00006

[B38] Berne CJ Donovan AJ White EJ Yellin AE Duodenal divericulization for duodenal and pancreatic injury Am J Surg 1974 127 503 507 4545015 10.1016/0002-9610(74)90305-5

[B39] Vaughan GD Frazier OH Graham DY Mattox KL Petmechy FF Jordan GL The use of pyloric exclusion in the management of severe duodenal injuries Am J Surg 1977 134 785 790 596547 10.1016/0002-9610(77)90325-7

[B40] Cukingnan RA Culliford AT Worth MH Surgical correction of a lateral duodenal fistula with the Roux-Y technique J Trauma 1975 15 519 523 805255 10.1097/00005373-197506000-00012

[B41] Klipfel AA Schein M Retroperitoneal perforation of the duodenum and necrotizing extension to the scrotum Surgery 2003 133 3 337 339 12660650 10.1067/msy.2003.42

[B42] Horvath K Freeny P Escallon J Heagerty P Comstock B Glickerman DJ Bulger E Sinanan M Langdale L Kolokythas O Safety and efficacy of video-assisted retroperitoneal debridement for infected pancreatic collections: a multicenter, prospective, single-arm phase 2 study Arch Surg 2010 145 9 817 825 20855750 10.1001/archsurg.2010.178

